# Antibacterial, Herbicidal, and Plant Growth-Promoting Properties of *Streptomyces* sp. STD57 from the Rhizosphere of *Adenophora stricta*

**DOI:** 10.3390/microorganisms12112245

**Published:** 2024-11-06

**Authors:** Dan He, Congting Gao, Shen Zhao, Hongmin Chen, Peng Li, Xishan Yang, Deping Li, Tingting Zhao, Hong Jiang, Chongxi Liu

**Affiliations:** 1 Heilongjiang Academy of Land Reclamation Sciences, Harbin 150030, China; hedanlqtm@163.com (D.H.); hellozhaoshen@163.com (S.Z.); bdhyxbjcc@163.com (X.Y.); liping10276@126.com (D.L.); 18249623297@163.com (T.Z.); jianghong0505@nefu.edu.cn (H.J.); 2 College of Plant Protection, Northeast Agricultural University, Harbin 150030, China; g2456692645@163.com (C.G.); chenhmvae@163.com (H.C.)

**Keywords:** antibacterial activity, herbicidal activity, plant growth-promoting activity, *Streptomyces* sp. STD57, *Ralstonia solanacearum*

## Abstract

Bacterial wilt triggered by the soil-borne pathogenic bacterium *Ralstonia solanacearum* is one of the most serious diseases in tomato plants, leading to huge economic losses worldwide. Biological control is considered an environmentally friendly and sustainable way to manage soil-borne diseases. In this study, *Streptomyces* sp. STD57 isolated from the rhizosphere of *Adenophora stricta* showed strong antibacterial activity against *R. solanacearum*. Pot experiments showed that strain STD57 exhibited a significant biocontrol effect (81.7%) on tomato bacterial wilt in the greenhouse environment. Furthermore, strain STD57 could inhibit the growth of weeds (*Amaranthus retroflexus*, *Portulaca oleracea*, and *Echinochloa crusgalli*) but promote the growth of crops (wheat, rice, and tomato). The plant growth-promoting substance was identified as indoleacetic acid (IAA) by high-pressure liquid chromatography–mass spectrometry and genome analysis. Coarse separation of the fermented extracts revealed that the antibacterial and herbicidal substances were mainly in the fermentation supernatant and belonged to different products. These findings suggested that strain STD57 may be a potential biocontrol and bioherbicide agent useful in agriculture.

## 1. Introduction

*Ralstonia solanacearum*, a Gram-negative bacterium dwelling in soil, is the causative agent of bacterial wilt disease in more than 250 plant species [1]. Its wide host range and global prevalence result in significant crop losses, particularly in tropical, subtropical, and warm temperate regions. This pathogen gains entry into hosts through wounded roots or sites of secondary root emergence. Once established, it rapidly spreads and proves challenging to manage [2]. Current disease management strategies involve the use of pesticides such as bismerthiazol, saisentong, and zinc thiazole. However, these methods pose serious environmental threats, contribute to pathogen resistance, and have detrimental effects on native microbial communities [3,4]. In light of these challenges, exploring biocontrol methods mediated by microorganisms presents a promising and sustainable approach to addressing the increasing agricultural demands.

The rhizosphere, defined as the region surrounding plant roots directly influenced by root secretions, plays a pivotal role in plant functioning [5]. Microbial activities within this zone are vital, facilitating nutrient uptake and safeguarding plants against pathogenic attacks [6]. Consequently, rhizobacteria have emerged as promising biocontrol agents. Biocontrol agents defend plants against biotic and abiotic stress by means of diverse mechanisms [7]. Direct biocontrol occurs through enhancing nutrients bioavailability and producing stimulating compounds. Indirect mechanisms involve the stimulation of plant innate immunity and direct antagonists through the production of antimicrobial substances. Among the most frequently employed microbial agents for controlling bacterial wilt are *Streptomyces* spp. [8,9], *Bacillus* spp. [10,11], and *Pseudomonas* spp. [12,13].

Actinomycetes, especially *Streptomyces* are known to exert a significant function in the plant rhizosphere through a diverse array of secondary metabolites related to plant growth promotion and phytopathogen control [14]. Strains belonging to the genus *Streptomyces* produce an array of natural products with high structural diversity including avermectin, blasticidin S, kasugamycin, zhongshengmycin, and validamycin, which have been widely used as agricultural antibiotics [15]. In addition, established symbiotic associations with crop plants, producing siderophores and phytohormones such as auxins, gibberellins, ethylene, and cytokinin-like chemicals, play pivotal roles in enhancing plant growth [16]. Commercial formulations based on *Streptomyces* have proven successful in controlling fungal phytopathogens. For instance, a biocontrol product derived from *Streptomyces* (*Streptomyces griseoviridis* K61) has been registered for application in Canada and numerous European countries, demonstrating efficacy against *Alternaria*, *Fusarium*, and *Phytophthora*. Another product, Actinovate^®^ (*Streptomyces lydicus* WYEC108), is advised for managing foliar and soil-borne fungal diseases [17].

In this study, we isolated and identified a rhizosphere *Streptomyces* from *Adenophora stricta*, which showed robust antagonistic activity against *R. solanacearum*. Subsequently, we conducted an in-depth examination of its efficacy as a biocontrol agent against tomato bacterial wilt caused by *R. solanacearum*. Additionally, we observed noteworthy herbicidal properties and plant growth-promoting effects associated with this isolate. To gain insights into its antibacterial, herbicidal, and plant growth-promoting mechanisms, we performed secondary metabolite isolation and genomic analysis, providing a preliminary understanding of the underlying processes.

## 2. Materials and Methods

### 2.1. Experimental Materials

Three weed seeds including *Amaranthus retroflexus*, *Portulaca oleracea*, and *Echinochloa crusgalli* were obtained from the College of Plant Protection, Northeast Agricultural University (Harbin, China). Wheat seed (Longmai 33) was obtained from the Crop Resources Institute of the Heilongjiang Academy of Agricultural Sciences (Harbin, China). Rice weed (Longjing 31) was kindly provided by the Heilongjiang Academy of Agricultural Sciences (Harbin, China). Tomato (Maofen 802) was obtained from the College of Horticulture and Landscape Architecture, Northeast Agricultural University (Harbin, China). *R. solanacearum* was kindly provided the Chinese Academy of Agricultural Sciences (Beijing, China).

### 2.2. Isolation and Identification of Strain STD57

Strain STD57 was obtained from the rhizosphere soil of *A. stricta* which was gathered in Yunnan Province, Southwest China. The rhizosphere soil sample was processed following previously established procedures [18]. Strain STD57 was isolated by employing the standard dilution plate method and cultivated on dulcitol proline agar (DPA) [19] supplemented with cycloheximide (50 mg/L) and nalidixic acid (20 mg/L). Following 14 days of aerobic incubation at 28 °C, the isolate was transferred and purified on ISP 3 agar [20]. Strain STD57 was characterized by morphology and phylogeny. Spore morphology was determined using a scanning electron microscope (Hitachi SU8010, Hitachi Co., Tokyo, Japan) after being cultured on ISP3 at 28 °C for 14 days. DNA extraction was conducted, followed by PCR amplification of the 16S rRNA gene sequence, as previously described [21]. A phylogenetic tree was generated using the neighbor-joining method [22] with Kimura’s two-parameter model [23] in Mega 7.0 [24]. The stability of the topology was evaluated using the bootstrap method with 1000 repetitions [25].

### 2.3. Activity Assessment of Strain STD57 Against R. solanacearum In Vitro

The antibacterial activity of strain STD57 against *R. solanacearum* was evaluated using the agar cylinders method. A 100 μL bacterial suspension (OD_600_ = 0.3) was spread onto nutrient agar (NA) [26]. Subsequently, a fresh agar plug (5 mm diameter, control group) or a mycelial agar plug from strain STD57 cultured on ISP3 agar for 7 days was placed on the NA plate, and then incubated at 37 °C. The diameter of the inhibition zone was measured 24 h post-incubation.

### 2.4. Biocontrol Efficacy of Strain STD57 Against R. solanacearum

In this study, a preventive experiment was conducted to assess the biocontrol efficacy of strain STD57 against *R. solanacearum* on tomatoes in a greenhouse. The spore suspension of strain STD57 was applied to the soil at the final concentrations of 10^5^ CFU/g, 10^6^ CFU/g, and 10^7^ CFU/g soil, respectively. Tomato seeds underwent surface sterilization using 70% ethanol for 1 min, followed by immersion in a 2% NaClO solution for 2 min. After washing with sterile water, the seeds were pre-germinated for 2 days at 25 °C before being transferred to a seedling tray and exposed to natural conditions. Upon reaching the four-leaf stage, the seedlings were transplanted singly into pots holding various concentrations of spores of strain STD57. The pots without strain STD57 were used as a negative control. After 1 week, a 15 mL suspension of *R. solanacearum* (OD_600_ = 0.3) was poured into the soil around the seedlings. There were 10 pots in each treatment, and the whole experiment was independently repeated three times. The experiment was conducted in a growth chamber which was set to mimic a 12 h photoperiod, maintaining a temperature of 37 °C and a humidity level of 75–90%. The duration of the experiment spanned 2 weeks. Disease severity was evaluated by using a five-class scale: 0 indicated no wilt, 1 represented 1–25% leaf wilt, 2 denoted 25–50% leaf wilt, 3 indicated 51–75% leaf wilt, and 4 denoted more than 75% leaf wilt. The Disease Index (DI) was calculated using the formula [Σ(number of plants with the same degree of disease severity × disease rating)/(maximum rating value × total number of plants)] × 100. Additionally, the biological control efficiency was computed using the formula [(DI of the negative control − DI of the treatment)/DI of the negative control] × 100% [9].

### 2.5. Activity Evaluation of Strain STD57 on Seed Germination of Weeds and Crops

The seeds of weeds (*A. retroflexus*, *P. oleracea* and *E. crusgalli*) and crops (wheat, rice and tomato) were surface sterilized with 2% NaClO for 20 min and washed 3 times with sterile water. Subsequently, the seeds were soaked with a spore suspension of strain STD57 at a concentration ranging from 10^6^ to 10^8^ CFU/mL. All the resultant seeds were incubated at 25 °C, and the lengths of shoots and roots were measured after 1 week. Each treatment comprised 30 seeds, and the entire experiment was replicated independently three times.

### 2.6. Effects of Strain STD57 on the Growth of Weeds and Crops in Pot Experiments

Two methods were used in the test. (1) Spore mixing with soil: the spore suspension of strain STD57 was applied to the soil at the final concentrations of 10^5^ CFU/g, 10^6^ CFU/g, and 10^7^ CFU/g soil, respectively. Subsequently, the sterilized seeds of weeds (*A. retroflexus* and *E. crusgalli*) and crops (wheat and tomato) were sown in the pots containing various concentrations of spores of strain STD57. After 4 weeks, the seedling fresh weight and dry weight were measured. (2) Foliar spray: After sterilization, the seeds of weeds and crops were sown in pots. After 3 weeks of cultivation, they were sprayed with spore suspension of strain STD57 at the concentration ranging from 10^5^ to 10^7^ CFU/mL. One week later, the seedling fresh weight and dry weight were recorded. The experiments were conducted in a growth chamber at 25 °C, with a 12 h light/12 h dark cycle. Each treatment comprised 6 pots, and the entire experiment was independently replicated three times.

### 2.7. Evaluation of Antibiotic Resistance of Strain STD57

The antibiotic resistance of strain STD57 was evaluated using an antimicrobial susceptibility test disks (BKMAM, Changde, China). The spore suspension (200 μL) was spread onto ISP3 agar. Subsequently, the disks with different antibiotics were placed on the plate, and then incubated at 28 °C for 3 days.

### 2.8. LC-MS Analysis of Spore Extracts of Strain STD57

Strain STD57 was cultured on an ISP3 plate for 2 weeks at 28 °C. The spores were extracted with 50 mL methanol and then evaporated in vacuo to dry and redissolved in 1 mL methanol. Samples (20 μL) were analyzed using an EasynLC1000 Ultra High-Performance Liquid Chromatography system (Thermo Fisher Scientific, Waltham, MA, USA) coupled with a QExactive mass spectrometer (Thermo Fisher Scientific, Waltham, MA, USA). For the UPLC analysis, mobile phases A (0.1% formic acid in water) and B (0.1% formic acid acetonitrile) were used. A gradient elution program was established as follows: 0–1.5 min, 95% A plus 5% B; 2.5 min, 90% A plus 10% B; 14 min, 60% A plus 40% B; 22–25 min, 5% A plus 95% B; 26–30 min, 95% A plus 5% B. A column temperature of 40 °C, injection volume of 2 μL, and flow rate of 0.25 μL/min were used.

### 2.9. Activity Evaluation of Fermentation Extracts of Strain STD57

Strain STD57 was initially cultured on an ISP3 plate for 2 weeks at 28 °C. Subsequently, it was inoculated into 250 mL baffled Erlenmeyer flasks which contained 50 mL of tryptone soy broth (TSB) and was cultivated for 5 days at 28 °C while shaking at 200 rpm. Following this, 3 mL of the culture was transferred into 250 mL baffled Erlenmeyer flasks filled with 50 mL of the production media and was cultivated at 28 °C for 7 days while shaking at 200 rpm. Five production media, PTM (maltodextrin 4%, lactose 4%, MOPS 2%, yeast extract 0.5%, pH 7.2–7.4), GYM (glucose 0.4%, malt extract 1%, soluble starch 2%, yeast extract 0.4%, CaCO_3_ 0.2%, pH 7.2–7.4), CG (cottonseed cake powder 2%, corn starch 3%, maltodextrin 2%, glucose 1%, yeast extract 0.5%, MgSO_4_·7H_2_O 0.1%, CaCO_3_ 0.2%, pH 7.2–7.4), MB (glucose 1%, yeast extract 0.2%, soluble starch 0.5%, tryptone 0.2%, NaCl 0.4%, K_2_HPO_4_ 0.05%, MgSO_4_·7H_2_O 0.05%, CaCO_3_ 0.2%, pH 7.2–7.4), and FG (fishmeal 1%, glycerol 2%, yeast extract 0.5%, CaCO_3_ 0.5%, pH 7.2–7.4), were selected for the fermentation. After fermentation, 50 mL of fermentation broth was centrifuged (4000 rev/min, 20 min) to obtain both supernatant and mycelia. The supernatant and mycelia were extracted with ethyl acetate and methanol, respectively. Subsequently, the extracts were evaporated under a vacuum to dryness and redissolved in methanol (2 mL). The antibacterial activity was assessed using the filter paper method. A bacterial suspension of 100 μL (OD_600_ = 0.3) was spread onto NA plates. Each filter paper (5 mm in diameter) saturated with 10 μL of the crude extract from either the supernatant or mycelia was positioned at the center of the NA plates and then incubated at 37 °C for 24 h. Additionally, herbicidal and plant growth-promoting activities were evaluated through seed germination experiments. Sterilized seeds were soaked in 100-fold-diluted crude extracts of the supernatant or mycelia for 1 week at 25 °C.

### 2.10. Separation of the Crude Extract of Strain STD57

Strain STD57 was initially cultured in 250 mL baffle Erlenmeyer flasks which contained 50 mL of TSB and was cultivated for 5 days at 28 °C while shaking at 200 rpm. After that, 18 mL of the culture was transferred into 1 L baffled Erlenmeyer flasks filled with 300 mL MB medium and was cultivated at 28 °C for 7 days while shaking at 200 rpm. The fermentation broth (20 L) was centrifuged at 4000 rev/min for 20 min, and the supernatant was extracted three times with ethyl acetate. The crude extract was evaporated under vacuum and then underwent column chromatography using a silica gel column (100–200 mesh). Elution was carried out by using mixtures of petroleum ether and ethyl acetate (10/1, 5/1, 1/1, 1/5, 1/10, and 0/1, *v*/*v*), and then by using mixtures of ethyl acetate and methanol (10/1, 5/1, 1/1, 1/5, and 1/10, *v*/*v*). These segments were evaporated under vacuum and redissolved in methanol, and then evaluated for their antibacterial and herbicidal activities.

### 2.11. Genome Analysis

The entire genome of strain STD57 was sequenced on the Illumina HiSeq PE150 platform (Illumina, San Diego, CA, USA) and assembled using SOAPdenovo 2. Genome mining for active secondary metabolites was performed with antiSMASH version 7.0 [27]. This Whole Genome Shotgun project has been deposited at DDBJ/ENA/GenBank under the accession JBFMIE000000000. The version described in this paper is version JBFMIE010000000.

### 2.12. Statistical Analysis

The data were analyzed by means of analysis of variance (ANOVA), followed by a Duncan’s multiple-range test with SPSS statistical software package (SPSS Inc., Cary, NC, USA, v.26).

## 3. Results

### 3.1. Isolation and Identification of Strain STD57 with Antagonistic Activity Against R. solanacearum

**Figure 1:**
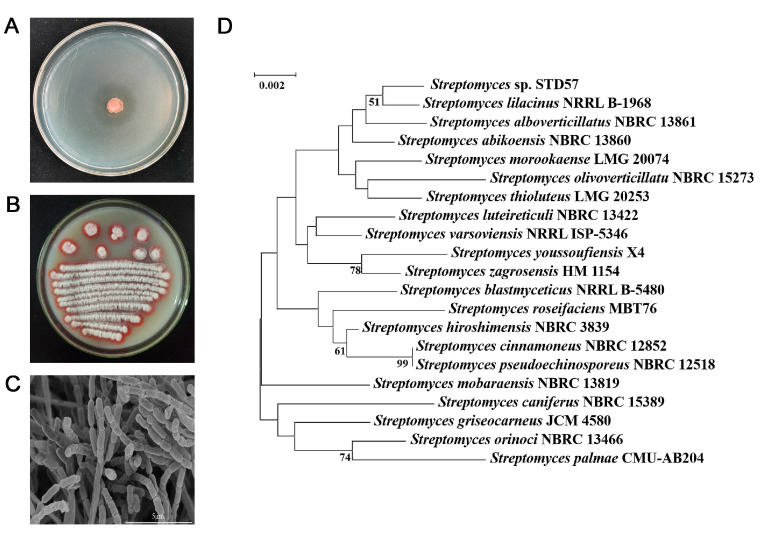
Characterization of strain STD57 with antagonistic activity against *R. solanacearum*. (**A**) The antagonistic activity of STD57 against *R. solanacearum*. (**B**) Colony morphology of STD57 grown on ISP3 medium for 2 weeks. (**C**) Scanning electron micrograph of STD57 grown on ISP3 agar for 2 weeks. Scale bar, 5 μm. (**D**) Phylogenetic tree constructed based on 16S rRNA gene sequences showing the relationship of STD57 with related taxa. Only bootstrap values greater than 50% (percentages of 1000 replications) are shown. Scale bar: 0.002 nucleotide substitutions per site.

Strain STD57 was isolated from the rhizosphere soil of *A. stricta*, which showed excellent antibacterial activity against *R. solanacearum*, with an inhibition diameter of 26.6 mm (Figure 1A). The morphology of strain STD57 exhibited typical characteristics of the genus *Streptomyces*, with white-colored aerial mycelium, rose-red substrate mycelium, and straight spore chains borne on the aerial mycelium when cultured on ISP3 medium at 28 °C for 2 weeks (Figure 1B,C). An analysis of the 16S rRNA sequence confirmed that strain STD57 belonged to the genus *Streptomyces*. It had the highest sequence identity to *Streptomyces lilacinus* NRRL B-1968^T^ (99.52%). A 16S rRNA-based phylogenetic tree showed that it formed a subclade with *S. lilacinus* NRRL B-1968^T^ (Figure 1D). Based on the above results, strain STD57 was classified as a species within the genus *Streptomyces*.

### 3.2. Biocontrol Effect of Strain STD57 on Tomato Bacterial Wilt

**Figure 2:**
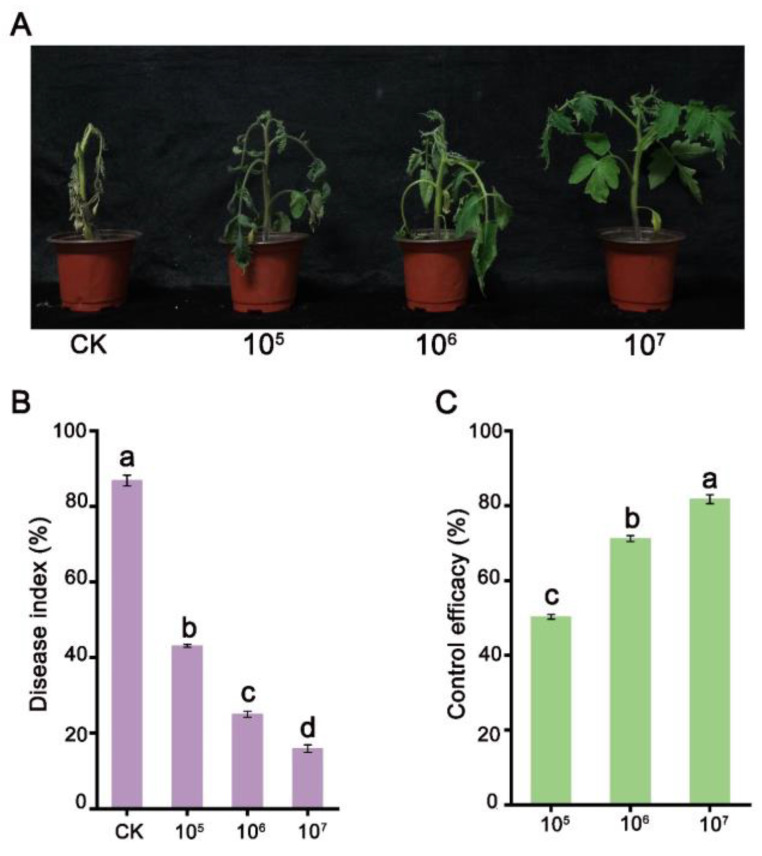
Effect of strain STD57 on tomato plants infected with *R. solanacearum*. (**A**) Disease symptoms of tomato bacterial wilt in the potted tomato plants inoculated with *R. solanacearum* and treated with varying spore concentrations of STD57. (**B**,**C**) Disease index and control efficacy were assessed 2 weeks after inoculation with *R. solanacearum* in tomato plants treated with STD57. Data with different lowercase letters denote significant differences at the 0.05 probability level.

To investigate whether strain STD57 had biocontrol potential against *R. solanacearum* in planta, we evaluated its efficacy in controlling tomato bacterial wilt on seedlings. Our findings demonstrated a significant reduction in the severity of the disease following the application of strain STD57, compared to the negative control group (Figure 2A). The disease index of treatment groups with spore concentrations of 10^5^ CFU/g, 10^6^ CFU/g, and 10^7^ CFU/g soil were 43.1%, 25.0%, and 15.9%, respectively, which were markedly lower than that of control group (86.8%) (Figure 2B). Notably, at a spore concentration of 10^7^ CFU/g in the soil, strain STD57 exhibited an impressive biocontrol efficacy of 81.7% (Figure 2C). This finding underscores the promising potential of strain STD57, particularly at this specific spore concentration, in managing tomato bacterial wilt effectively.

### 3.3. Herbicidal Activity of Strain STD57

**Figure 3:**
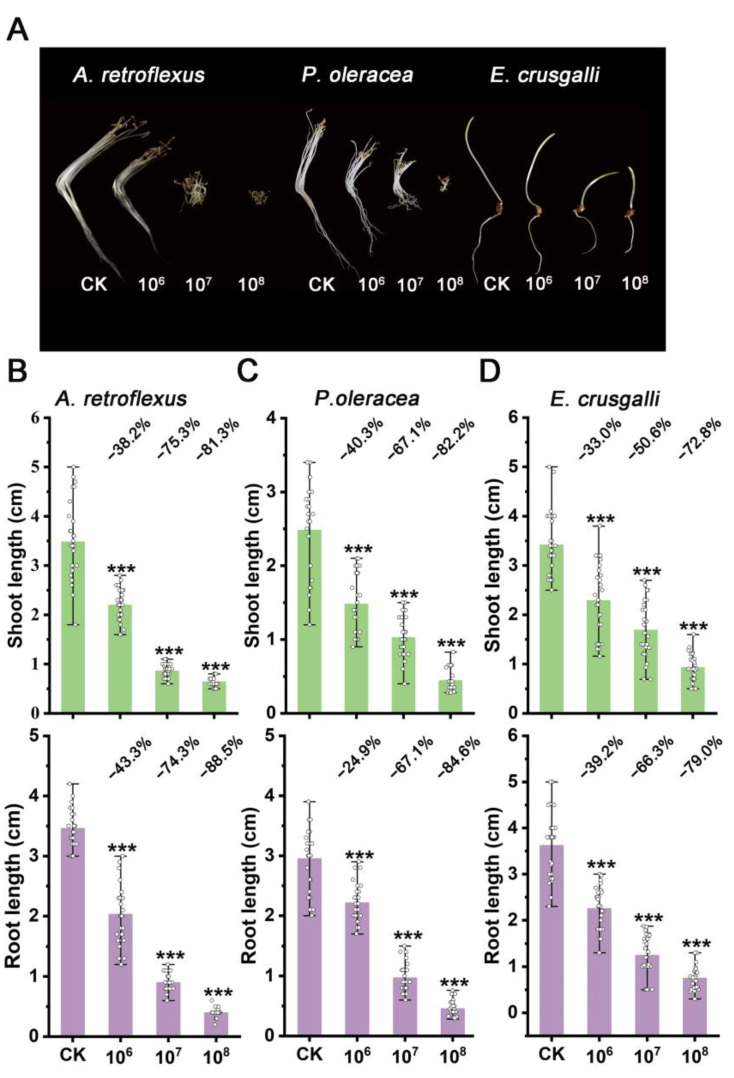
Preemergence inhibition activities of strain STD57 at different spore concentrations on weeds. (**A**) Inhibition effects of STD57 at different spore concentrations on different weeds. (**B**) Inhibition rates of STD57 at different spore concentrations on shoot and root length of *A. retroflexus*. (**C**) Inhibition rates of STD57 at different spore concentrations on the shoot and root length of *P. oleracea*. (**D**) Inhibition rates of STD57 at different spore concentrations on the shoot and root length of *E. crusgalli*. *** *p* < 0.001.

To fully explore the agricultural potential of strain STD57, we tested its herbicidal activity using three common weed species: *A. retroflexus*, *P. oleracea*, and *E. crusgalli*. Seed germination experiments were conducted with spore suspensions. The results showed that strain STD57 did not inhibit the germination rates of the weed seeds, but had a noticeable inhibitory effect on the growth of shoots and roots (Figure 3A). As the spore concentration increased, the inhibitory effect also intensified. When the spore concentration was 10^8^ CFU/mL, the shoot lengths of *A. retroflexus*, *P. oleracea,* and *E. crusgalli* were decreased by 81.3%, 82.2%, and 72.8%, respectively, while the root lengths were decreased by 88.5%, 84.6%, and 79.0%, respectively (Figure 3B–D).

**Figure 4:**
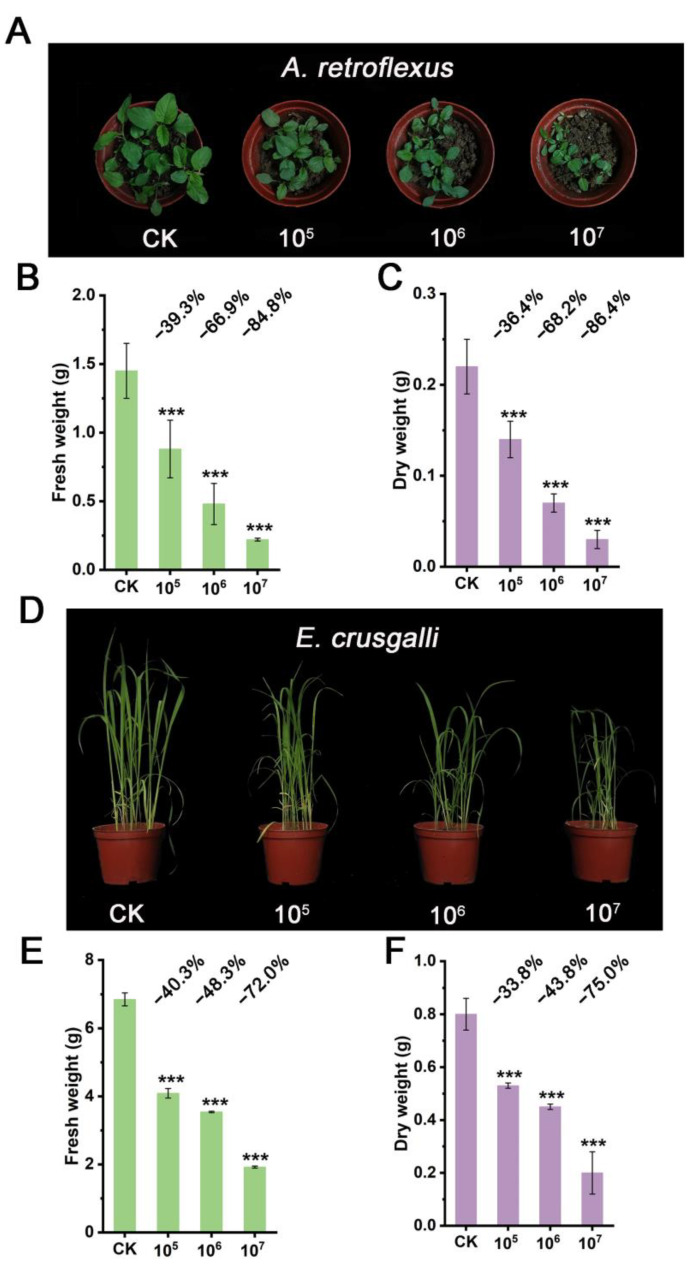
Postemergence inhibition activities of strain STD57 at different spore concentrations on weeds after mixing spores with soil. (**A**) Inhibiting effects of STD57 at different spore concentrations on *A. retroflexus*. (**B**,**C**) Inhibition rates of STD57 at different spore concentrations on fresh and dry weight of *A. retroflexus*. (**D**) Inhibiting effects of STD57 at different spore concentrations on *E. crusgalli*. (**E**,**F**) Inhibition rates of STD57 at different spore concentrations on fresh and dry weight of *E. crusgalli*. *** *p* < 0.001.

To further confirm the herbicidal activity of this strain, we conducted pot experiments using *A. retroflexus* and *E. crusgalli* as test weeds. Two methods, spore mixing with soil and foliar spray, were employed for testing. The results showed similar effects for both methods. Herbicidal activity was positively correlated with spore concentration (Figure 4A,B and Figure S1A,B). At a spore concentration of 10^7^ CFU/mL or 10^7^ CFU/g in soil, compared to the control group, *A. retroflexu* exhibited a reduction of more than 80% in both fresh weight and dry weight (Figure 4C,D and Figure S1C,D), while *E. crusgalli* showed reductions of over 70% in fresh weight and over 60% in dry weight (Figure 4E,F and Figure S1E,F). These results indicate the potential of this strain to be developed into a bioherbicide.

### 3.4. Plant Growth-Promoting Effect of Strain STD57

**Figure 5:**
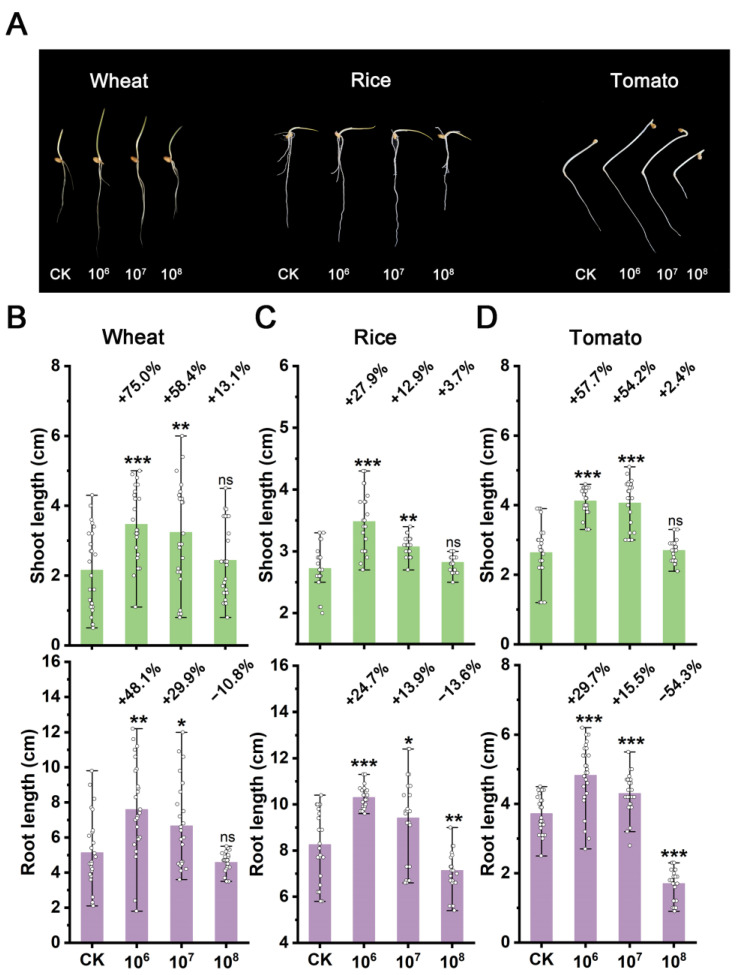
Preemergence growth-promoting activities of strain STD57 at different spore concentrations on crops. (**A**) Growth-promoting effects of STD57 at different spore concentrations on different crops. (**B**) Growth-promoting rates of STD57 at different spore concentrations on shoot and root length of wheat. (**C**) Growth-promoting rates of STD57 at different spore concentrations on the shoot and root length of rice. (**D**) Growth-promoting rates of STD57 at different spore concentrations on the shoot and root lengths of tomato. ns, no significance; * *p* < 0.05; ** *p* < 0.01; *** *p* < 0.001.

To evaluate the safety of strain STD57 on crops, we investigated its effects on the seed germination of wheat, rice, and tomato. When the spore concentration was 10^8^ CFU/mL, it exhibited inhibitory effects on the root growth of all three crops (Figure 5A). Surprisingly, at spore concentrations of 10^7^ and 10^6^ CFU/mL, strain STD57 had a significant stimulating effect on both the root and shoot growth of the three crops. The most prominent promotion occurred at a spore concentration of 10^6^ CFU/mL, where the root lengths of wheat, rice, and tomato increased by 48.1%, 24.7%, and 29.7%, respectively, and shoot lengths increased by 75.0%, 27.9%, and 57.7%, respectively (Figure 5B–D).

**Figure 6:**
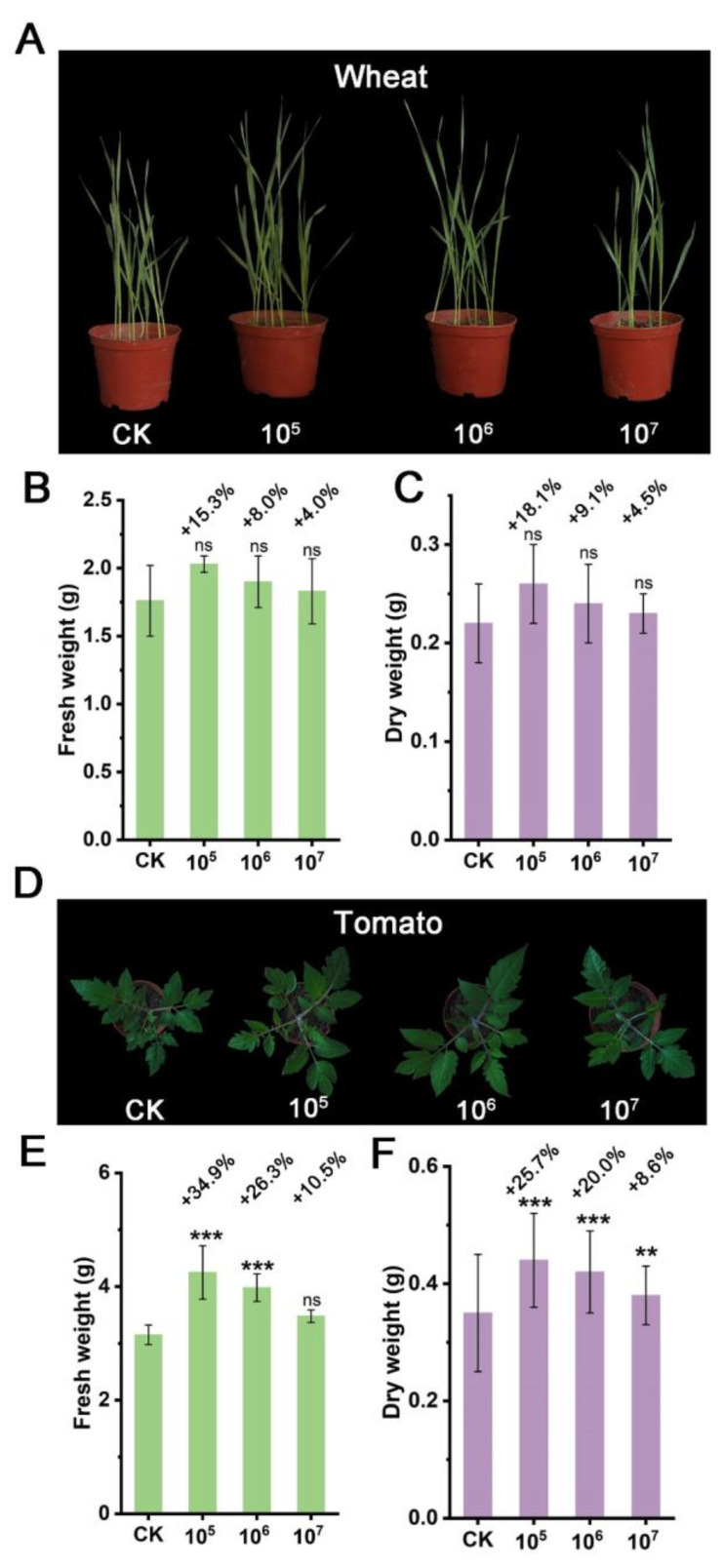
Postemergence growth-promoting activities of strain STD57 at different spore concentrations on weeds by spore mixing with soil. (**A**) Growth-promoting effects of STD57 at different spore concentrations on wheat. (**B**,**C**) Growth-promoting rates of STD57 at different spore concentrations on fresh and dry weight of wheat. (**D**) Growth-promoting effects of STD57 at different spore concentrations on tomato. (**E**,**F**) Growth-promoting rates of STD57 at different spore concentrations on fresh and dry weight of tomato. ns, no significance; ** *p* < 0.01; *** *p* < 0.001.

To confirm the plant growth-promoting activity of this strain, wheat and tomato were chosen as test crops for pot experiments. Foliar spore spraying did not exhibit a significant growth-promoting activity on both crops (Figure S2A–F). However, the spore mixed with soil method showed that strain STD57 had a significant growth-promoting effect on tomato plants. The optimal spore concentration for growth promotion was 10^5^ CFU/g in soil, resulting in a 34.9% increase in fresh weight and a 25.7% increase in dry weight compared to the control group (Figure 6A–F).

### 3.5. Evaluation of Antibiotic Resistance of Strain STD57

In this study, a total of 30 antibiotics were selected to test the resistance of strain STD57. The results showed that the strain was resistant to most antibiotics, but was also sensitive to 11 antibiotics, including clarithromycin, vancomycin, sulfamethoxa, chloramphenicol, amikacin, gentamycin, kanamycin, streptomycin, erythromycin, azithromycin, and piperacillin (Figure S3 and Table S1). This result provides guidance for the future application of the strain in the environment.

### 3.6. Property of Strain STD57 Active Substances

**Figure 7:**
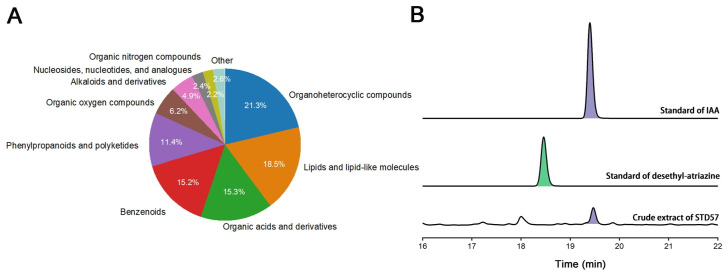
LC-MS and HPLC analysis of extracts from STD57. (**A**) LC-MS analysis. (**B**) HPLC analysis.

Because the spore suspension of strain STD57 showed good antibacterial, herbicidal, and plant growth-promoting activities, we analyzed the spore extracts using high-pressure liquid chromatography–mass spectrometry (LC-MS). The results revealed that this strain can produce abundant metabolites, which mainly belong to organoheterocyclic compounds, lipids and lipid-like molecules, organic acids and derivatives, benzenoids phenylpropanoids and polyketides, and so on (Figure 7A and Table S2). IAA, a plant hormone with plant growth-promoting activity produced by many bacteria, was found in the metabolites. The HPLC analysis further confirmed that strain STD57 produced IAA (Figure 7B). Desethyl-atrazine, a metabolite of the herbicide atrazine, was also detected in the metabolites. However, the HPLC analysis indicated that this strain did not yield this product (Figure 7B). In addition, we found no natural metabolites associated with antibacterial activity.

To further determine the active components of strain STD57, five fermentation media, including PTM, GYM, MB, CG and FG, were used to detect secondary metabolites of the strain. The results showed that all five fermentation products showed good antibacterial and herbicidal activities. Among them, the fermentation product from the MB medium had the best herbicidal activity towards three weeds and caused a >70% biomass reduction (shoot length and root length), and showed the best inhibition effect against *R. solanacearum* (Figure S4). Furthermore, we found that the antibacterial and herbicidal active components of strain STD57 were mainly present in the supernatant extract, not in the mycelial extract (Figure S5). Studies of STD57 active substances localization and a silica gel column chromatography analysis indicated that the antibacterial activities were in the petroleum ether/ethyl acetate segment (1/1, 1/5, 1/10, and 0/1) (Figure S6), while the herbicidal activities were mainly present in the ethyl acetate/methanol segment (10/1, 5/1, and 1/1) (Figure S7). This result suggests that strain STD57 produced different secondary metabolites for antibacterial and herbicidal effects. However, unfortunately, we have yet to determine the active secondary metabolites.

### 3.7. Mining the Biosynthetic Potential of Strain STD57

Data obtained from antiSMASH analysis showed that STD57 encodes fifty-six secondary metabolite biosynthesis gene clusters (BGCs), including eight non-ribosomal peptide synthetases (NRPSs), five type I polyketide synthases (T1PKSs), five terpenes, five siderophores, five lanthipeptides, four lassopeptides, four ribosomally synthesized and posttranslationally modified peptides (RiPPs), four polyene macrolides, two type III polyketide synthases (T3PKS), two nonaketides, one hybrid non-ribosomal peptide synthetase/polyketide synthase (NRPS+PKS), one hydrogen-cyanide, one type II polyketide synthase (T2PKS), onepolyyne, one hybrid nucleotide/polyketide, one pyrroloindole, one spiroaminals, one lipopeptide, one octaketide, one butyrolactone, one ectoine, and one crocagin (Table S3). Sixteen putative gene clusters showed a high similarity (the similarity of the core biosynthetic genes > 60%) to known guanipiperazine, hopene, marieosin, paenibactin, qinichelins, kirromycin, toxoflavin, eurocidin, EDHA, antipain, ectoine, AmfS, lagmysin, filipin, griseobactin, and jawsamycin gene clusters. Among them, four shared 100% similarity with the accession sequence, which encode antipain, ectoine, lagmysin, and jawsamycin. This observation indicates that strain STD57 has the potential to synthesize numerous secondary metabolites. Subsequently, we investigated the indole-3-acetamide pathway (IAM)-mediated IAA synthetic pathway, which has been identified in the genus *Streptomyces* [28]. *IaaM* and *IaaH*, encoding tryptophan monooxygenase and indole-3-acetamide hydrolase, are critical for IAA biosynthesis in the IAM pathway. Blastp analysis showed that two adjacent genes (GM001596 and GM001595) in the STD57 genome shared 89.24% and 64.23% identities with IaaM and IaaH from *Streptomyces coelicolor* A3(2), respectively. This result demonstrates the existence of IAM-mediated IAA biosynthesis in strain STD57.

## 4. Discussion

In most agroecosystems, soilborne plant pathogens pose a significant challenge to achieving optimal marketable yields [29]. Unlike pathogens attacking above-ground plant parts, soilborne pathogens prove more resistant to management and control measures. These pathogens are adept at thriving in the bulk soil, with the rhizosphere serving as the primary infection site where the pathogen establishes a parasitic relationship upon encountering the plant. It is within the rhizosphere that the intricate community of microflora and microfauna can engage with the pathogen, influencing the outcome of the infection process. Consequently, improving the suppressiveness of the rhizosphere soil emerges as a crucial strategy to mitigate disease incidence while preserving plant health and sustaining agricultural production [30]. For example, Wang et al. has reported that the application of bio-organic fertilizers, incorporating an antagonistic microorganism, contributed to suppressing soil-borne fungi and bacterial pathogen *R. solanacearum* invasion into the rhizosphere [9]. In this study, we aimed to screen rhizosphere actinomycetes as potential biocontrol agents for the management of tomato bacterial wilt. Our finding revealed that *Streptomyces* sp. STD57 exhibited strong inhibitory activity against *R. solanacearum*. The disease control effects of STD57 showed significant dose–effect dependence. When a spore concentration of 10^7^ CFU/g in soil was applied, the disease control efficacy exceeded 80%. This effect may be due to the inhibitory impact of STD57 on *R. solanacearum*, which requires the accumulation of adequate antibacterial substances.

Actinomycetes represent a substantial and widely distributed cohort of soil microorganisms, accounting for approximately 10 to 50% of the soil microflora community. Many researchers have underscored their significance as prolific producers of secondary metabolites [31,32]. The metabolites produced exhibit diverse biological activities and functions, including antifungal, antibacterial, insecticidal, and herbicidal properties. Among rhizosphere microbes, actinomycetes hold a distinctive position in promoting plant growth due to their array of beneficial traits [14]. The majority of studied actinomycetes species known to promote plant growth also display antibacterial or antifungal properties, a characteristic highlighted during their screening as biocontrol agents [16]. This assertion is supported by the development of various products such as Mycostop^®^ (*Streptomyces griseoviridis* K61, Bayer, Leverkusen, Germany), Actinovate^®^ (*Streptomyces lydicus* WYEC108, Novozymes, Copenhagen, Denmark), and Nogall^®^ (*Agrobacterium radiobacter* K1026, Bayer, Leverkusen, Germany). However, it is rarely reported that biocontrol agents based on actinomycetes have the potential to promote plant growth and control weeds. In this study, we found that strain STD57 exhibited multiple biological functions, including antibacterial, herbicidal, and plant growth-promoting activities, and was most effective at a spore concentration of 10^7^ CFU/g in soil. Therefore, strain STD57 has the potential to be developed into a biological control agent, which can be applied for tomato bacterial wilt control and as a bioherbicide.

Strains belonging to the genus *Streptomyces* have been reported to be producers of several plant hormones, such as auxin, cytokinin-like chemicals, gibberellins, ethylene, salicylic acid, jasmonic acid, and abscisic acid, which are crucial for promoting plant growth and also for assisting plants in dealing with biotic and abiotic stresses [33,34]. To identify the active substances, we analyzed the spore extracts via LC-MS and identified the plant-growth-promoting substance as IAA, a plant hormone belonging to the auxin class. The biosynthesis pathway of IAA via IAM has received extensive attention. In this pathway, tryptophan is initially transformed into IAM by tryptophan monooxygenase, and then the IAM is hydrolyzed to IAA and ammonia by indole-3-acetamide hydrolase. In microorganisms, two crucial genes, namely IaaM and IaaH, which encode tryptophan monooxygenase, and indole-3-acetamide hydrolase, respectively, were first identified in *Pseudomonas savastanoi* [28]. Additionally, the IAM pathway has also been recognized as the main pathway for IAA biosynthesis in the genus *Streptomyces* [28]. Genetic analysis has screened out homologous genes of *IaaM* and *IaaH*, suggesting the existence of the IAM pathway in strain STD57. Furthermore, we fermented the strain and determined MB as the optimal fermentation medium. Despite multiple attempts, we have yet to determine the antibacterial and herbicidal substances. However, what we can confirm is that they belong to distinct products. Further research on the antibacterial and herbicidal substances of strain STD57 needs to be conducted.

New bioinformatics technologies and methods, especially genome sequencing, can help achieve more accurate classification of target strains and offer deeper understandings of their secondary metabolism and other functions. Subsequent genomic analysis indicates that strain STD57 is rich in secondary metabolites. To fully recognize the biosynthetic potential of these secondary metabolites, the genome of strain STD57 was sequenced and annotated. Fifty-six BGCs were predicted, which may be accountable for producing known or unknown secondary metabolites. Only four gene clusters exhibited 100% similarity with known BGCs, which are responsible for the biosynthesis of antipain, ectoine, lagmysin, and jawsamycin, respectively. Jawsamycin has been reported to possess antifungal activity [35]. Nevertheless, strain STD57 did not exhibit antifungal activity. It is speculated that this gene cluster may be silenced in strain STD57. Most of the secondary metabolites produced by strain STD57 are still unknown. From this, it can be inferred that the secondary metabolites synthesized by strain STD57 contain unknown active components, which are worthy of further exploration.

## 5. Conclusions

In this study, *Streptomyces* sp. STD57 isolated from the rhizosphere of *A. stricta* showed strong inhibitory activity against *R. solanacearum*. In pot experiments, the strain not only exhibited a good biocontrol effect on tomato bacterial wilt, but also could control weeds and promote plant growth. The plant growth-promoting activity of this strain was determined to be associated with IAA production, but the antimicrobial and herbicidal substances remained unknown. The significant antibacterial and herbicidal activity of this strain imply its use in agriculture as a potent biocontrol agent and bioherbicide.

## Data Availability

The original contributions presented in the study are included in the article/Supplementary Materials, further inquiries can be directed to the corresponding author/s.

## Supplementary Materials

The following supporting information can be downloaded at: https://www.mdpi.com/article/10.3390/microorganisms12112245/s1, Figure S1: Postemergence inhibition activities of strain STD57 at different spore concentrations on weeds by foliar spray; Figure S2: Postemergence growth-promoting activities of strain STD57 at different spore concentrations on weeds by foliar spray; Figure S3: The herbicidal and antibacterial activities of five fermentation products; Figure S4: The antibacterial and herbicidal activities of MB fermentation product; Figure S5: The antibacterial activity of petroleum ether/ethyl acetate segment (1/1, 1/5, 1/10, and 0/1) against *R. solanacearum*; Figure S6: The herbicidal activity of ethyl acetate/methanol segment (10/1, 5/1, and 1/1) on *A. retroflexus*; Figure S7: The herbicidal activity of ethyl acetate/methanol segment (10/1, 5/1, and 1/1) on *A. retroflexus*; Table S1: LC-MS analysis of spore extracts; Table S2: Secondary metabolite clusters in STD57; Table S3: Secondary metabolite clusters in strain STD57.
