# Supplementary material for: Antibacterial, Herbicidal, and Plant Growth-Promoting Properties of Streptomyces sp. STD57 from the Rhizosphere of Adenophora stricta
Source: Microorganisms. 2024 Nov 6;12(11):2245. doi: 10.3390/microorganisms12112245 (PMC11596161; doi:10.3390/microorganisms12112245)
Supplement: Supplementary file 1 [file microorganisms-12-02245-s001.zip › Supplementary file.pdf]

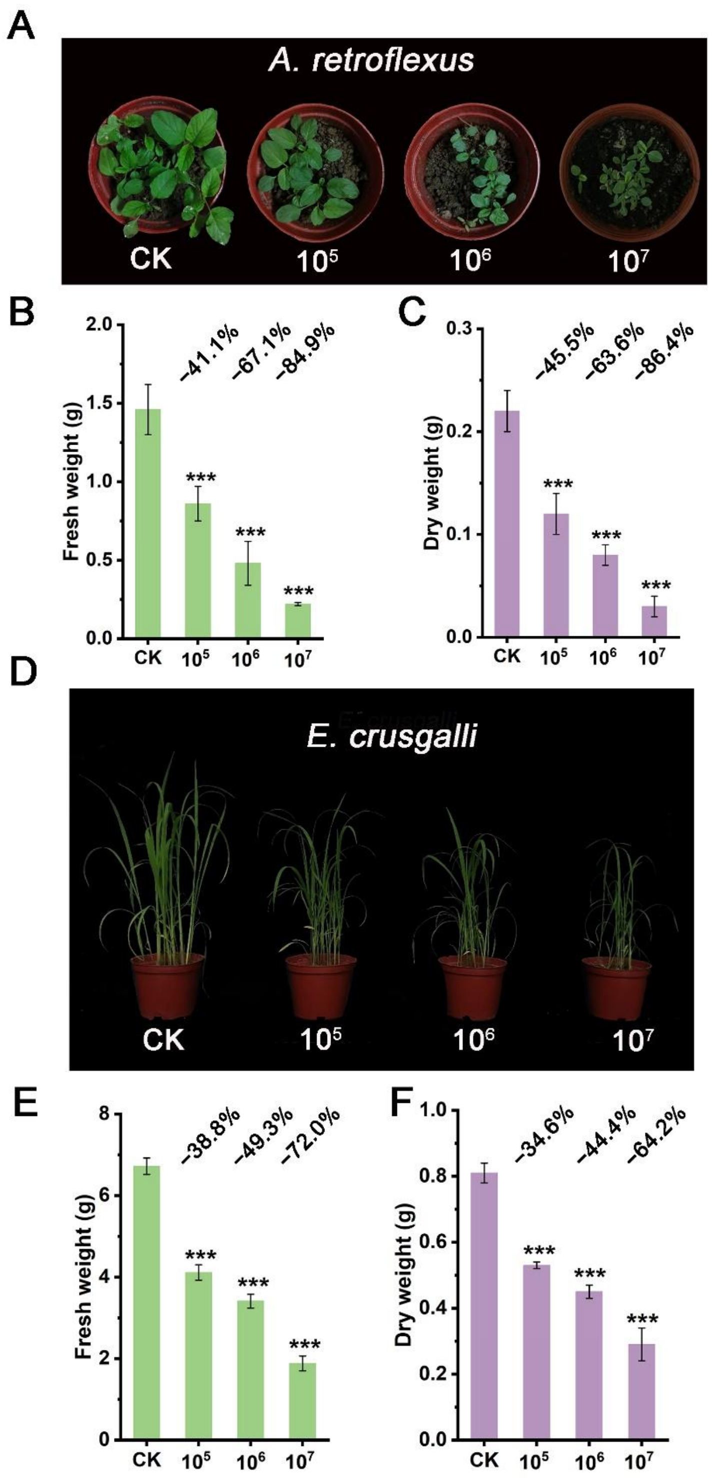

Figure S1. Postemergence inhibition activities of strain STD57 at different spore concentrations on weeds by foliar spray. A, Inhibition effects of STD57 at different spore concentrations on *A. retroflexus*. B and C, Inhibition rates of STD57 at different spore concentrations on fresh and dry weight of *A. retroflexus*. D, Inhibition effects of STD57 at different spore concentrations on *E. crusgalli*. E and F, Inhibition rates of STD57 at different spore concentrations on fresh and dry weight of *E. crusgalli*. ***$P < 0.001$.

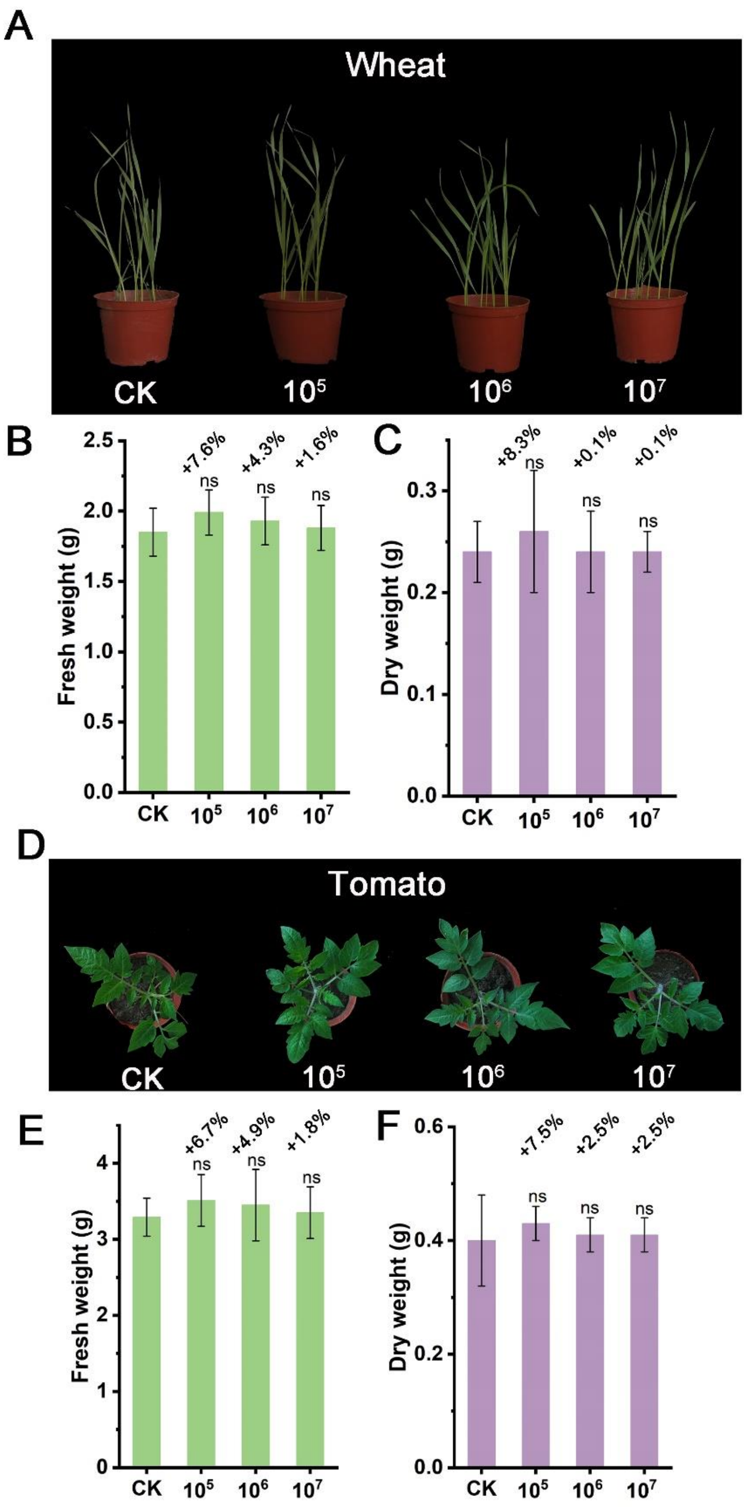

Figure S2. Postemergence growth-promoting activities of strain STD57 at different spore concentrations on weeds by foliar spray. A, Growth-promoting effects of STD57 at different spore concentrations on wheat. B and C, Growth-promoting rates of STD57 at different spore concentrations on fresh and dry weight of wheat. D, Growth-promoting effects of STD57 at different spore concentrations on tomato. E and F, Growth-promoting rates of STD57 at different spore concentrations on fresh and dry weight of tomato. ns, no significance.

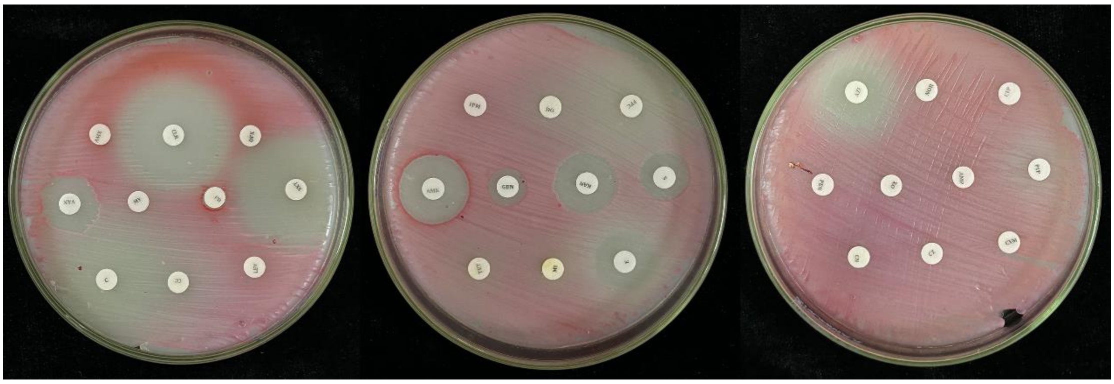

Figure S3. Antibiotic resistance evaluation of strain STD57. AMX, amoxicillin; CLR, clarithromycin; OFX, ofloxacin; VAN, vancomycin; MY, lincomycin; PB, polymyxin B; SXT, sulfamethoxa; C, chloramphenicol; CC, clindamycin; LEV, levofloxacin; IPM, imipenem; DO, doxycycline; FFC, florfenicol; AMK, amikacin; GEN, gentamycin; KAN, kanamycin; S, streptomycin; TET, tetracycline; MI, minocycline; E, erythromycin; AZI, azithromycin; NOR, norfloxacin; CIP, ciprofloxacin; PEN, penicillin; OX, oxacillin; AMP, ampicillin; PIP, piperacillin; CN, cephalexin; CZ, cefamezin; CXM, cefuroxime.

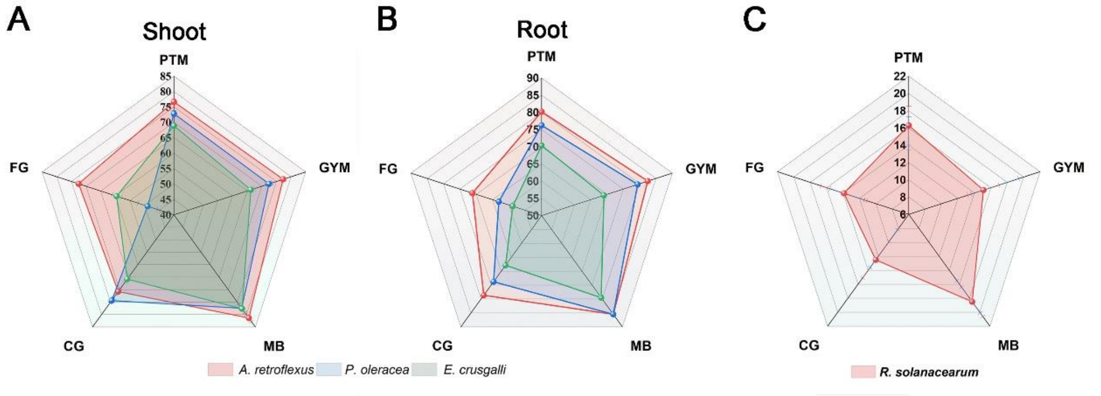

Figure S4. The herbicidal and antibacterial activities of five fermentation products. A, Inhibition rates of five fermentation products on shoot length of three weeds. B, Inhibition rates of five fermentation products on root length of three weeds. C, Inhibition diameters of five fermentation products against *R. solanacearum*.

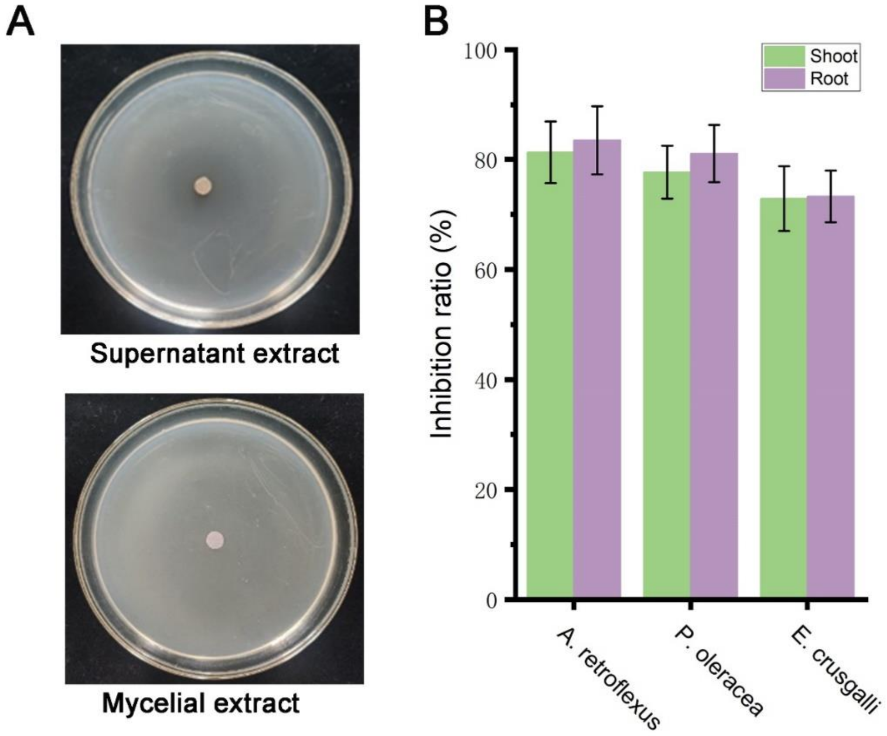

Figure S5. The antibacterial and herbicidal activities of MB fermentation product. A, Antibacterial effect of supernatant and mycelial extracts against *R. solanacearum*. Inhibition rates of five fermentation products on shoot length of three weeds. B, Inhibition ratio of supernatant extract on shoot and root length of three weeds.

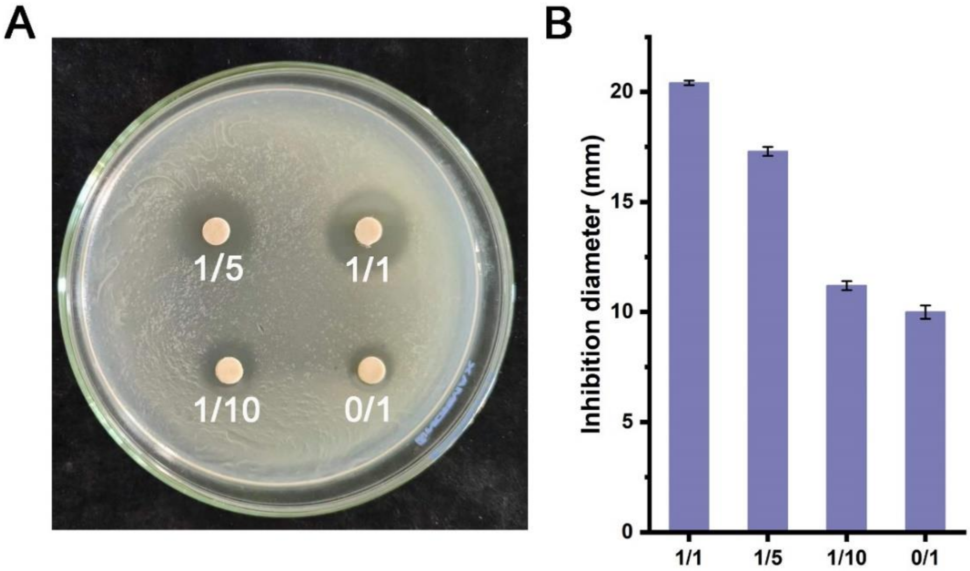

Figure S6. The antibacterial activity of petroleum ether/ethyl acetate segment (1/1, 1/5, 1/10, and 0/1) against *R. solanacearum*.

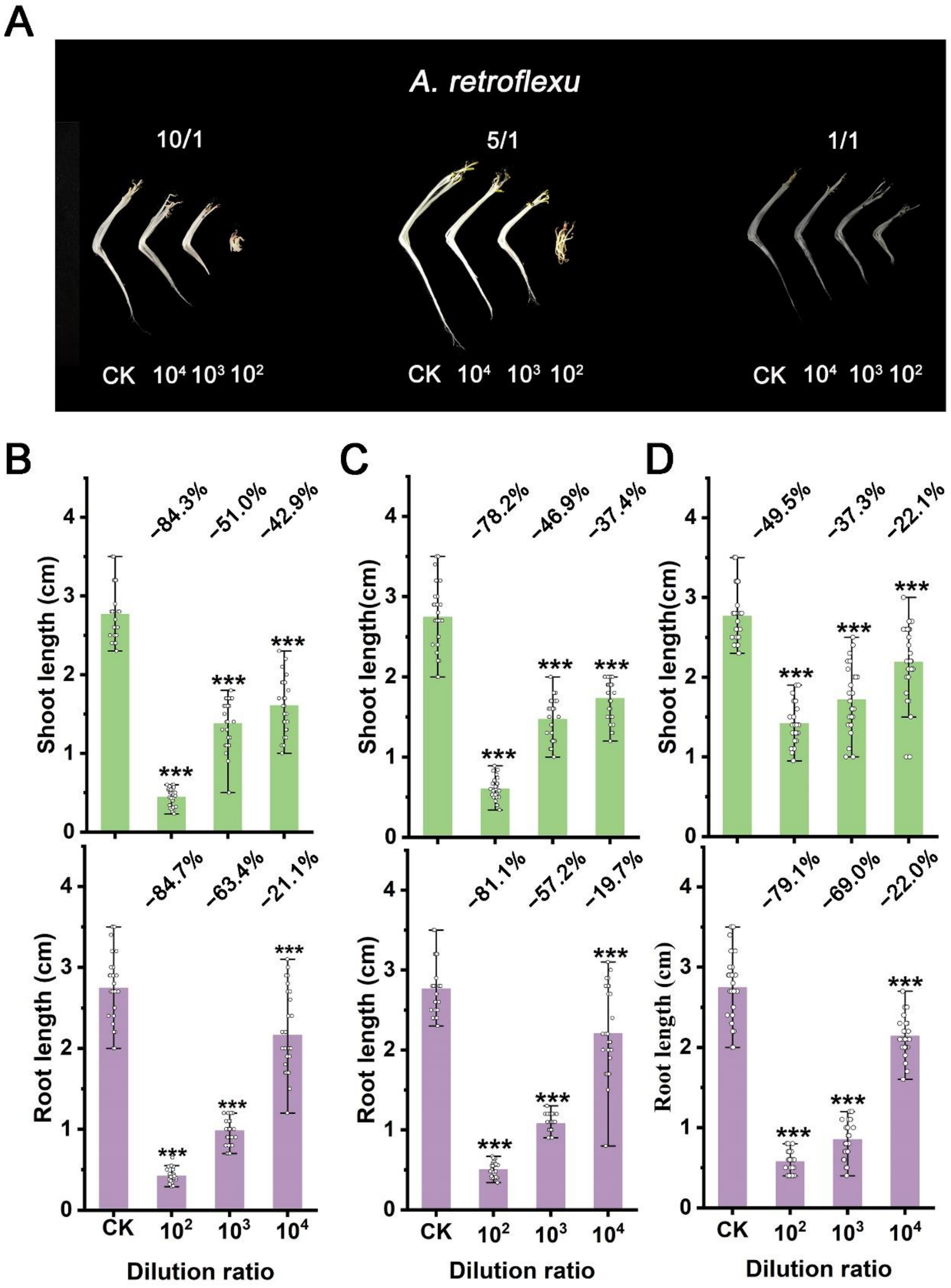

Figure S7. The herbicidal activity of ethyl acetate/methanol segment (10/1, 5/1, and 1/1) on *A. retroflexus*. A, Inhibition effects of different dilution ratio of ethyl acetate/methanol segment on *A. retroflexus*. B, Inhibition rates of different dilution ratio of ethyl acetate/methanol segment (10/1) on shoot and root length of *A. retroflexus*. C, Inhibition rates of different dilution ratio of ethyl acetate/methanol segment (5/1) on shoot and root length of *A. retroflexus*. D, Inhibition rates of different dilution ratio of ethyl acetate/methanol segment (1/1) on shoot and root length of *A. retroflexus*.

Table S1. Antibiotic resistance evaluation of strain STD57. +, resistant; -, sensitive

Antibiotic	Content per disc	STD57
Amoxicillin	25 μg	+
Clarithromycin	15 μg	-
Ofloxacin	5 μg	+
Vancomycin	30 μg	-
Lincomycin	2 μg	+
Polymyxin B	300 IU	+
Sulfamethoxa	25 μg	-
Chloramphenicol	30 μg	-
Clindamycin	2 μg	+
Levofloxacin	5 μg	+
Imipenem	10 μg	+
Doxycycline	30 μg	+
Florfenicol	30 μg	+
Amikacin	30 μg	-
Gentamycin	10 μg	-
Kanamycin	30 μg	-
Streptomycin	10 μg	-
Tetracycline	30 μg	+
Minocycline	30 μg	+
Erythromycin	15 μg	-
Azithromycin	15 μg	-
Norfloxacin	10 μg	+
Ciprofloxacin	5 μg	+
Penicillin	10 U	+
Oxacillin	1 μg	+
Ampicillin	10 μg	+
Piperacillin	100 μg	-
Cephalexin	30 μg	+
Cefamezin	30 μg	+
Cefuroxim	30 μg	+

Table S3. Secondary metabolite clusters in strain STD57.

Cluster	Type	Region	Most similar known cluster	Similarity
Cluster 1	T3PKS	Scaffold 1: 80,611-121,720	vazabitide A	28%
Cluster 2	lassopeptide	Scaffold 1: 231,071-253,615	TVA-YJ-2	19%
Cluster 3	octaketide	Scaffold 2: 47,068-80,216	kinamycin	19%
Cluster 4	lipopeptide	Scaffold 2: 281,475-303,810	enduracidin	10%
Cluster 5	polyene macrolide	Scaffold 2: 435,991-471,072	hexacosalactone A	13%
Cluster 6	RiPP	Scaffold 3: 273,206-307,423	siomycin A	7%
Cluster 7	siderophore	Scaffold 4: 1-20,047	peucechelin	20%
Cluster 8	NRPS	Scaffold 5:1-25,672	guanipiperazine	80%
Cluster 9	terpene	Scaffold 5: 116,281-171,127	hopene	76%
Cluster 10	spiroaminals	Scaffold 5: 181,086-227,679	marineosin	90%
Cluster 11	siderophore	Scaffold 5: 292,483-341,277	paenibactin	83%
Cluster 12	siderophore	Scaffold 6: 178,600-292,536	qinichelins	88%
Cluster 13	butyrolactone	Scaffold 7: 21,585-32,814	—	—
Cluster 14	crocagin	Scaffold 7: 58,096-86,119	—	—
Cluster 15	NRPS+PKS	Scaffold 7: 96,943-260,678	kirromycin	76%
Cluster 16	nucleotide derivative	Scaffold 7:269,858-315,655	toxoflavin	64%
Cluster 17	T1PKS	Scaffold 8: 1-104,629	eurocidin	60%
Cluster 18	terpene	Scaffold 8: 138,709-160,874	sarasinoside	18%
Cluster 19	NRPS	Scaffold 9: 1-101,093	cadaside	14%
Cluster 20	RiPP	Scaffold 9: 150,280-161,317	—	—
Cluster 21	siderophore	Scaffold 9: 194,911-208,451	EDHA	88%
Cluster 22	lanthipeptide	Scaffold 10: 202,146-224,854	—	—
Cluster 23	NRPS	Scaffold 12: 20,042-63,103	kitacinnamycin	9%
Cluster 24	NRPS	Scaffold 12: 85,241-133,460	antipain	100%
Cluster 25	T1PKS	Scaffold 12: 189,031-233,149	heme D1	11%
Cluster 26	ectoine	Scaffold 13: 23,253-33,651	ectoine	100%
Cluster 27	NRPS	Scaffold 14: 210,467-220,862	saframycin	4%
Cluster 28	terpene	Scaffold 15: 29,584-50,786	—	—
Cluster 29	NRPS	Scaffold 15: 57,739-108,507	dudomycin	13%
Cluster 30	RiPP	Scaffold 15: 197,379-208,269	—	—
Cluster 31	lanthipeptide	Scaffold 16: 57,701-84,174	AmfS	80%
Cluster 32	pyrroloindole	Scaffold 17: 23,324-82,983	mitomycin	52%
Cluster 33	T1PKS	Scaffold 17: 136,881-188,459	hexacosalactone A	11%
Cluster 34	RiPP	Scaffold 17: 205,032-216,234	—	—
Cluster 35	hydrogen-cyanide	Scaffold 19: 51,370-64,545	cosmomycin C	5%
Cluster 36	lanthipeptide	Scaffold 20: 97,479-122,444	—	—
Cluster 37	lassopeptide	Scaffold 22: 56,160-78,806	lagmysin	100%
Cluster 38	lanthipeptide	Scaffold 23: 6,940-45,921	SSV-2083	27%
Cluster 39	polyene macrolide	Scaffold 23: 61,780-140,449	filipin	92%
Cluster 40	NRPS	Scaffold 24: 1-38,709	—	—
Cluster 41	nonaketide	Scaffold 26: 14,551-57,709	azicemicin B	15%
Cluster 42	nonaketide	Scaffold 27: 54,676-97,834	azicemicin B	15%
Cluster 43	polyyne	Scaffold 31: 1-16,794	cepacin A	37%
Cluster 44	siderophore	Scaffold 31: 19,590-66,819	griseobactin	61%
Cluster 45	lassopeptide	Scaffold 32: 5,919-19224	aborycin	21%
Cluster 46	T1PKS	Scaffold 32: 21,871-70213	ansaseomycin	13%
Cluster 47	terpene	Scaffold 33: 14,343-35,500	legonindolizidine	8%
Cluster 48	T3PKS	Scaffold 36: 6,711-53,190	lagriene	4%
Cluster 49	nucleotide+polyketide	Scaffold 40: 1-54,897	jawsamycin	100%
Cluster 50	T1PKS	Scaffold 43: 1-47,170	notonesomycin A	15%
Cluster 51	lanthipeptide	Scaffold 45: 25,961-43,483	—	—
Cluster 52	polyene macrolide	Scaffold 47: 1-35,902	pepticinnamin E	12%
Cluster 53	polyene macrolide	Scaffold 48: 1-35,379	pentamycin	53%
Cluster 54	terpene	Scaffold 54: 5,785-26,792	valinomycin	13%
Cluster 55	T2PKS	Scaffold 62: 1-17,307	saprolmycin E	36%
Cluster 56	lassopeptide	Scaffold 63: 1-16,463	—	—
